# Supplementary material for: Faa1 membrane binding drives positive feedback in autophagosome biogenesis via fatty acid activation
Source: J Cell Biol. 2024 Apr 4;223(7):e202309057. doi: 10.1083/jcb.202309057 (PMC10993510; doi:10.1083/jcb.202309057)
Supplement: Table S1 — shows yeast strains used in this study. [file JCB_202309057_TableS1.docx]

Table 1 Yeast strains used in this study

| Name | Genotype | Background | Reference |
| --- | --- | --- | --- |
| BY4741 | MAT a; *hi3Δ1 leu2Δ0 met15Δ0 ura3Δ0* |  | Euroscarf |
| W303 | ade2-1; leu2-3; his3-11, 15; trp1-1; ura3-1; can1-100 |  | Thomas and Rothstein (1989) |
| SMY1 |  | BY4741 | Euroscarf |
| SMY276 | MAT a; *pep4::NAT ATG9-EGFP-TAP:URA* | BY4741 | Sawa-Makarska et al. (2020) |
| SMY462 | *faa1::KANMX4* | BY4741 | Euroscarf |
| SMY464 | *faa1::KANMX4 leu2::GAL1pr-FAA1-mCherry-TAP-ADH1ter:LEU2* | BY4741 | This study |
| SMY465 | *faa1::KANMX4 leu2::GAL1pr-FAA1-EGFP-TAP-ADH1ter:LEU2* | BY4741 | This study |
| SMY503 | *faa1::KANMX4 leu2::GAL1pr-faa1-K387D/K388D/K636D/K647D-EGFP-TAP-ADH1ter:LEU2* | BY4741 | This study |
| SMY506 | *faa1::KANMX4 leu2::GAL1pr-faa1-K387D/K388D/K612D/K619D/K636D/K647D-EGFP-TAP-ADH1ter:LEU2* | BY4741 | This study |
| SMY511 | *Δatg19::HIS3 pRS304-prATG8-mCherry-ATG8 FAA1-3xyEGFP-CaURA3*∆faa4*::natMX6*∆faa3*::kanMX6* | W303 | Schütter et al. (2020) |
| SMY512 | *Δatg19::HIS3 pRS304-prATG8-mCherry-ATG8 Faa1- K387D/K388D/K636D/K647D -3xyEGFP-CaURA3* ∆faa4*::natMX6*∆faa3*::kanMX6* | W303 | This study |
| SMY513 | *Δatg19::HIS3 pRS304-prATG8-mCherry-ATG8 Faa1-K387D/K388D/K612D/K619D/K636D/K647D -3xyEGFP-CaURA3* ∆faa4*::natMX6*∆faa3*::kanMX6* | W303 | This study |
| SMY514 | *Δatg19::natMX6 Δfaa1::FAA1-kanMX6 Δfaa3::TRP1 Δfaa4::HIS3 pRS306-prATG8-2xyEGFP-ATG8* | W303 | Schütter et al. (2020) |
| SMY515 | *Δatg19::natMX6 Δfaa1::Faa1- K387D/K388D/K636D/K647D-kanMX6 Δfaa3::TRP1 Δfaa4::HIS3 pRS306-prATG8-2xyEGFP-ATG8* | W303 | This study |
| SMY516 | *Δatg19::natMX6 Δfaa1::Faa1-K387D/K388D/K612D/K619D/K636D/K647D -kanMX6 Δfaa3::TRP1 Δfaa4::HIS3 pRS306-prATG8-2xyEGFP-ATG8* | W303 | This study |
| SMY517 | *Δatg19::natMX6 Δfaa1::TRP1 Δfaa3::kanMX6 Δfaa4::HIS3 pRS306-prATG8-2xyEGFP-ATG8* | W303 | This study |
| SMY518 | *faa1::KANMX4 leu2::GAL1pr-faa1- K387D/K388D/K636D/K647D-mCherry-TAP-ADH1ter:LEU2* | W303 | This study |
| SMY520 | *faa1::KANMX4 leu2::GAL1pr-faa1-K387D/K388D/K636D/K647D-10xHis-TAP-ADH1ter:LEU2* | BY4741 | This study |
| SMY523 | *faa1::KANMX4 leu2::GAL1pr-FAA1-10xHis-TAP-ADH1ter:LEU2* | BY4741 | This study |
| SMY524 | *pRS304-prATG8-mCherry-ATG8 Faa1-yEGFP-FYVE-CaURA3* ∆faa4*::natMX6*∆faa3*::kanMX6* | W303 | This study |
| SMY525 | *pRS304-prATG8-mCherry-ATG8 FAA1- K387D/K388D/K636D/K647D-yEGFP-FYVE-CaURA3*∆faa4*::natMX6*∆faa3*::kanMX6* | W303 | This study |
