# Supplementary material for: Faa1 membrane binding drives positive feedback in autophagosome biogenesis via fatty acid activation
Source: J Cell Biol. 2024 Apr 4;223(7):e202309057. doi: 10.1083/jcb.202309057 (PMC10993510; doi:10.1083/jcb.202309057)
Supplement: Table S2 — shows plasmids used in this study. [file JCB_202309057_TableS2.docx]

Table S2 Plasmids used in this study

| Name | Expressing | Vector | Expression system | Reference |
| --- | --- | --- | --- | --- |
| SMC1181 | ProteinA-TEV-Atg14-Atg6-Vps34-Vps15 | pBig2ab | Sf9 cells | Sawa-Makarska et al. (2020) |
| SMC1230 | 6xHis-TEV-Atg9-mEGFP-2xStrep | pFastBac HT | Sf9 cells | Sawa-Makarska et al. (2020) |
| SMC1496 | FadD-TEV-6xHis | pET-Duet | *E. coli* Rosetta pLysS | This study |
