# Supplementary material for: Faa1 membrane binding drives positive feedback in autophagosome biogenesis via fatty acid activation
Source: J Cell Biol. 2024 Apr 4;223(7):e202309057. doi: 10.1083/jcb.202309057 (PMC10993510; doi:10.1083/jcb.202309057)
Supplement: Table S3 — shows lipids used in this study. [file JCB_202309057_TableS3.docx]

Table S3 Lipids used in this study

|  | Name | Supplier | Catalog number | Concentration |
| --- | --- | --- | --- | --- |
| POPC | 1-palmitoyl-2-oleoyl-glycero-3-phosphocholine | Avanti Polar Lipids, Inc. | 850457C | 10 mg/mL |
| POPS | 1-palmitoyl-2-oleoyl-sn-glycero-3-phospho-L-serine (sodium salt) | Avanti Polar Lipids, Inc. | 840034C | 10 mg/mL |
| POPE | 1-palmitoyl-2-oleoyl-sn-glycero-3-phosphoethanolamine | Avanti Polar Lipids, Inc. | 850757 | 10 mg/mL |
| Liver PI | L-α-phosphatidylinositol (Liver, Bovine) (sodium salt) | Avanti Polar Lipids, Inc. | 840042C | 10 mg/mL |
| PI3P | 1,2-dioleoyl-sn-glycero-3-phospho-(1'-myo-inositol-3'-phosphate) (ammonium salt) | Avanti Polar Lipids, Inc. | 850150P | 1 mg/mL |
| PI4P | 1,2-dioleoyl-sn-glycero-3-phospho-(1'-myo-inositol-4'-phosphate) (ammonium salt) | Avanti Polar Lipids, Inc. | 850151P | 1 mg/mL |
| Lissamine rhodamine-DHPE | Lissamine™ rhodamine B 1,2dihexadecanoyl-sn-glycero-3phosphoethanolamine, triethylammonium salt | Invitrogen | L-1392 | 1 mg/mL |
| NBD-DPPE | 1,2-dipalmitoyl-sn-glycero-3-phosphoethanolamine-N-(7-nitro-2-1,3-benzoxadiazol-4-yl) (ammonium salt) | Avanti Polar Lipids, Inc. | 810144C | 1 mg/mL |
| ATTO 390-DOPE | ATTO390-labelled 1,2-Dioleoyl-sn-glycero3-phosphoethanolamine | ATTO-Tec | AD390-161 | 10 mg/mL |
| DGS-NTA | 1,2-dioleoyl-sn-glycero-3-[(N-(5-amino-1-carboxypentyl)iminodiacetic acid)succinyl] (nickel salt) | Avanti Polar Lipids, Inc. | 790404C | 5 mg/mL |
