# Supplementary material for: Faa1 membrane binding drives positive feedback in autophagosome biogenesis via fatty acid activation
Source: J Cell Biol. 2024 Apr 4;223(7):e202309057. doi: 10.1083/jcb.202309057 (PMC10993510; doi:10.1083/jcb.202309057)
Supplement: Table S4 — shows liposomes prepared for this study. [file JCB_202309057_TableS4.docx]

Table S4 Liposomes prepared for this study

| Figure | Name | Composition; % (w/v) |
| --- | --- | --- |
| Fig. 1B | Atg9 vesicle-like LPs + PI3P | 42.5 % POPC, 6 % POPS, 6 % POPE, 41.5 % liver PI, 2.5 % PI3P, 1.5 % ATTO 390-DOPE |
|  | POPC only | 98.5 % POPC, 1.5 % ATTO 390-DOPE |
| Fig. 2A and 2B | Atg9-like LPs + 2.5 % PI3P | 44 % POPC, 6 % POPS, 41.5 % liver PI, 5.5 % POPE, 2.5 % PI3P, 0.5 % lissamine rhodamine-DHPE |
|  | Atg9-like LPs w/o PI3P | 44 % POPC, 6 % POPS, 44 % liver PI, 5.5 % POPE, 0.5 % lissamine rhodamine-DHPE |
|  | 25 % PI | 63 % POPC, 6 % POPS, 25 % liver PI, 5.5 % POPE, 0.5 % lissamine rhodamine-DHPE |
|  | 5 % PI | 83 % POPC, 6 % POPS, 5 % liver PI, 5.5 % POPE, 0.5 % lissamine rhodamine-DHPE |
|  | POPC only | 99.5 % POPC, 0.5 % lissamine rhodamine-DHPE |
| Fig. 2C and S1B | POPC + 17.5 % POPS + 5 % PI3P | 77 % POPC, 17.5 % POPS, 5 % PI3P, 0.5 % lissamine rhodamine-DHPE |
|  | POPC + 17.5 % POPS + 5 % PI4P | 77 % POPC, 17.5 % POPS, 5 % PI4P, 0.5 % lissamine rhodamine-DHPE |
|  | POPC + 27.5 % POPS w/o PIP | 72 % POPC, 27.5 % POPS, 0.5 % lissamine rhodamine-DHPE |
|  | POPC + 17.5 % POPS w/o PIP | 82 % POPC, 17.5 % POPS, 0.5 % lissamine rhodamine-DHPE |
| Fig. 3C and 3D | Atg9-like LP + PI3P | 44 % POPC, 6 % POPS, 41.5 % liver PI, 5.5 % POPE, 2.5 % PI3P, 0.5 % lissamine rhodamine-DHPE |
| Fig. 4B | Atg9-like LPs + 2.5 % PI3P | 44 % POPC, 6 % POPS, 41.5 % liver PI, 6 % POPE, 2.5 % PI3P |
|  | Atg9-like LPs w/o PI3P | 44 % POPC, 6 % POPS, 44 % liver PI, 6 % POPE |
|  | POPC LPs | 100 % POPC |
| Fig. 4C and S3A | Atg9-like LPs + 2.5 % PI3P | 44 % POPC, 6 % POPS, 41.5 % liver PI, 6 % POPE, 2.5 % PI3P |
| Fig. 5A | Atg9-like LPs + 2.5 % PI3P | 4 % POPC, 6 % POPS, 2 % POPE, 44 % liver PI, 2 % NBD-DPPE and 2 % lissamine rhodamine-DHPE |
| Fig. 5B and S3E | Atg9-like LPs + 5 % DGS-NTA | 39 % POPC, 6 % POPS, 41.5 % liver PI, 6 % POPE, 2.5 % PI3P, 5 % DGS-NTA |
|  | Atg9-like LPs w/o DGS-NTA | 44 % POPC, 6 % POPS, 41.5 % liver PI, 6 % POPE, 2.5 % PI3P |
|  | POPC LPs + 5 % DGS-NTA | 95 % POPC, 5 % DGS-NTA |
|  | POPC LPs w/o DGS-NTA | 100 % POPC |
| Fig. S1A | 40 % POPS | 59.5 % POPC, 40 % POPS, 0.5 % lissamine rhodamine-DHPE |
|  | 20 % POPS | 79.5 % POPC, 20 % POPS, 0.5 % lissamine rhodamine-DHPE |
|  | 10 % POPS | 89.5 % POPC, 10 % POPS, 0.5 % lissamine rhodamine-DHPE |
|  | 0 % POPS | 99.5 % POPC, 0.5 % lissamine rhodamine-DHPE |
| Fig. S1C | Atg9-like LP + 44 % PI | 44 % POPC, 6 % POPS, 44 % liver PI, 5.5 % POPE, 0.5 % lissamine rhodamine-DHPE |
|  | Atg9-like LP + 25 % PI | 63 % POPC, 6 % POPS, 25 % liver PI, 5.5 % POPE, 0.5 % lissamine rhodamine-DHPE |
|  | POPC only LPs | 99.5 % POPC, 0.5 % lissamine rhodamine-DHPE |
