# Supplementary figures and images for: Faa1 membrane binding drives positive feedback in autophagosome biogenesis via fatty acid activation
Source: J Cell Biol. 2024 Apr 4;223(7):e202309057. doi: 10.1083/jcb.202309057 (PMC10993510; doi:10.1083/jcb.202309057)

B)

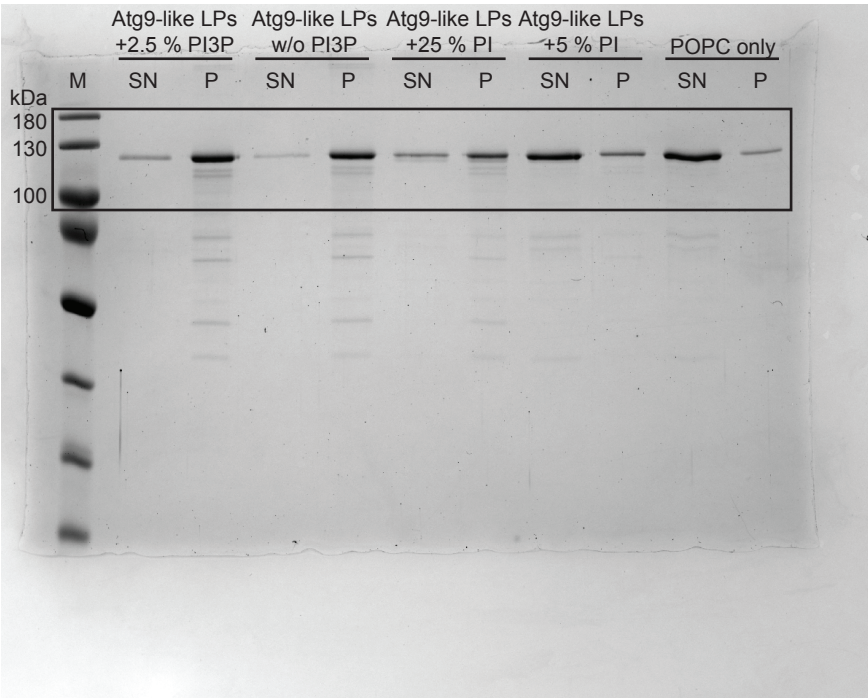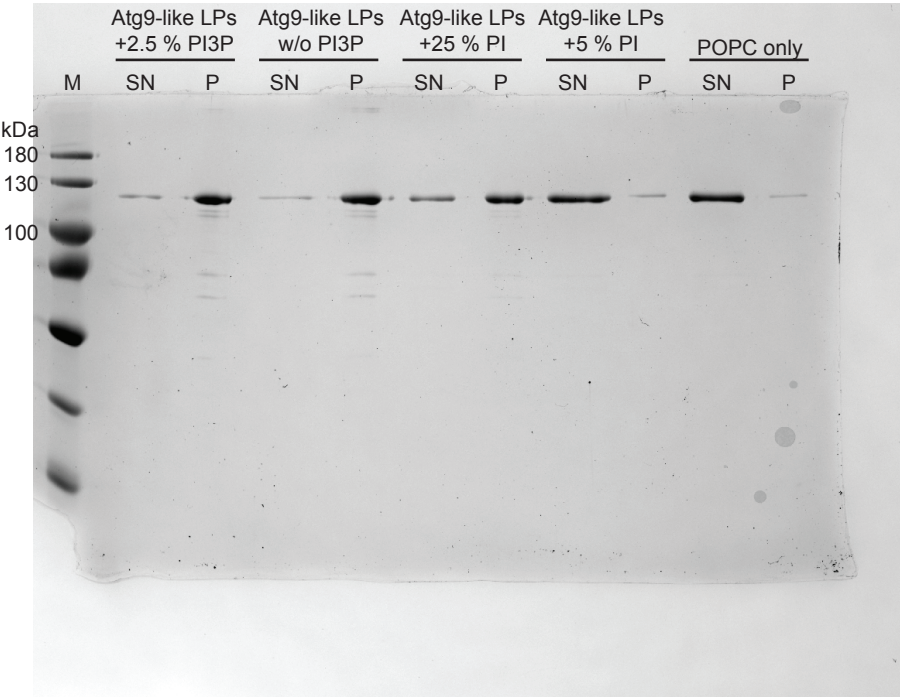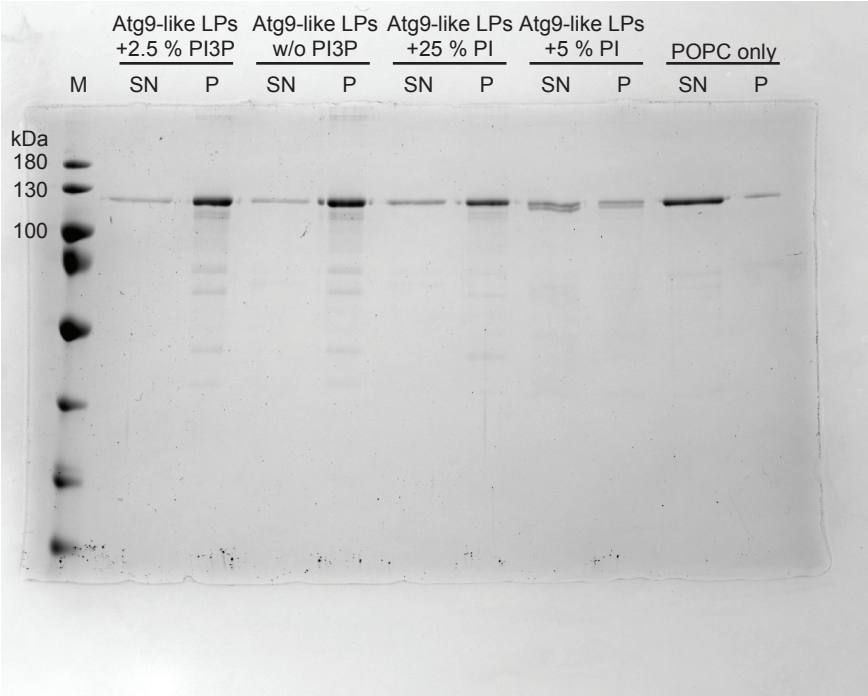

C)

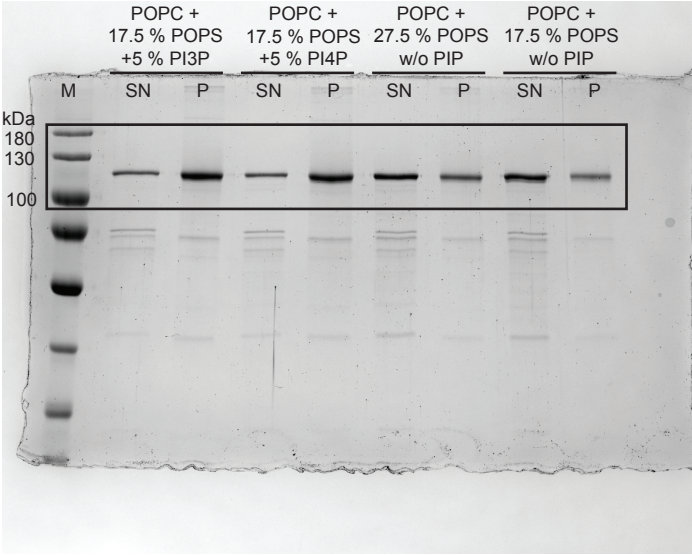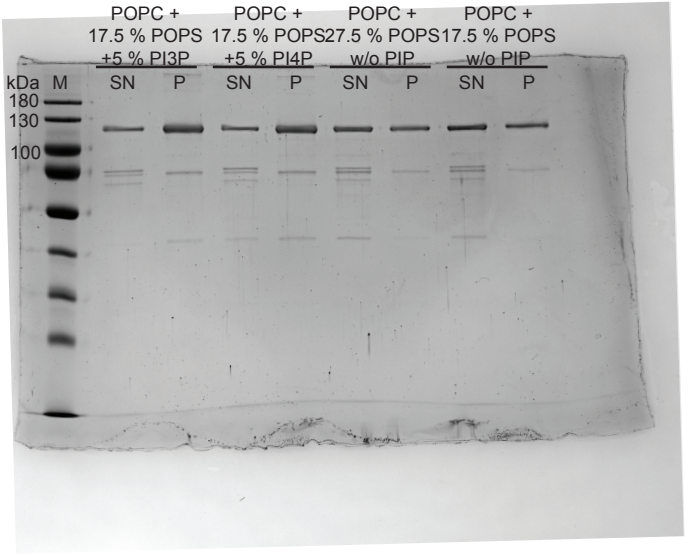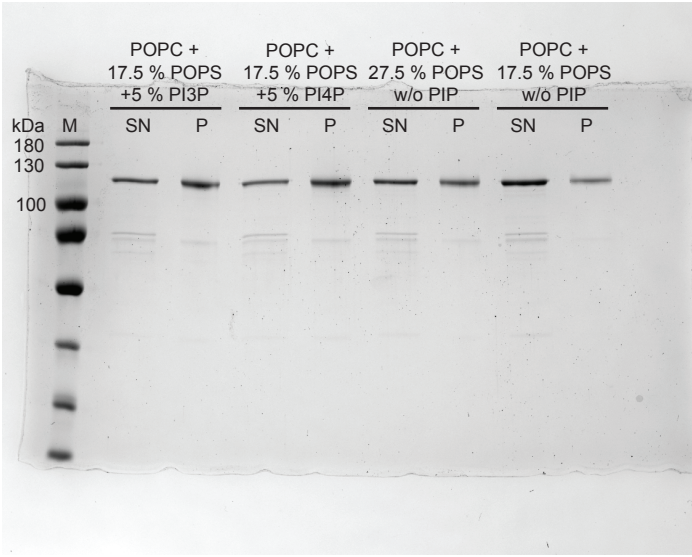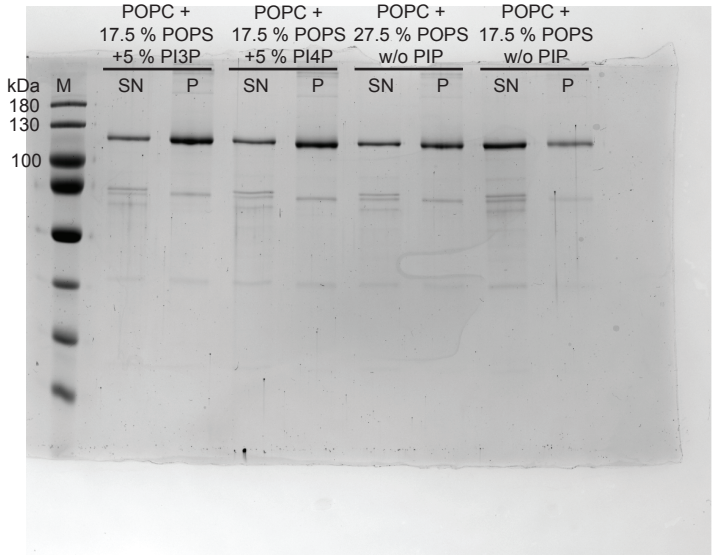

Supplement: SourceData F2 — is the source file for Fig. 2. [file JCB_202309057_SourceDataF2.pdf]

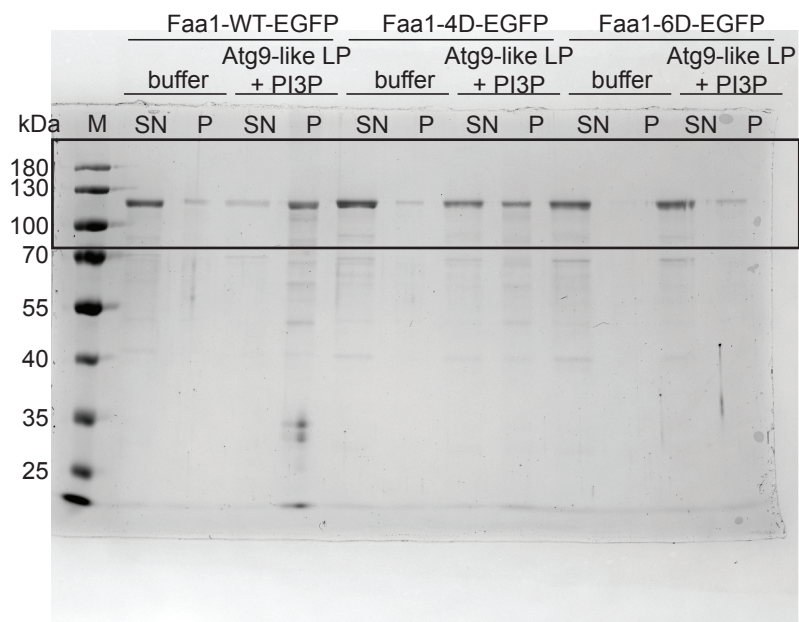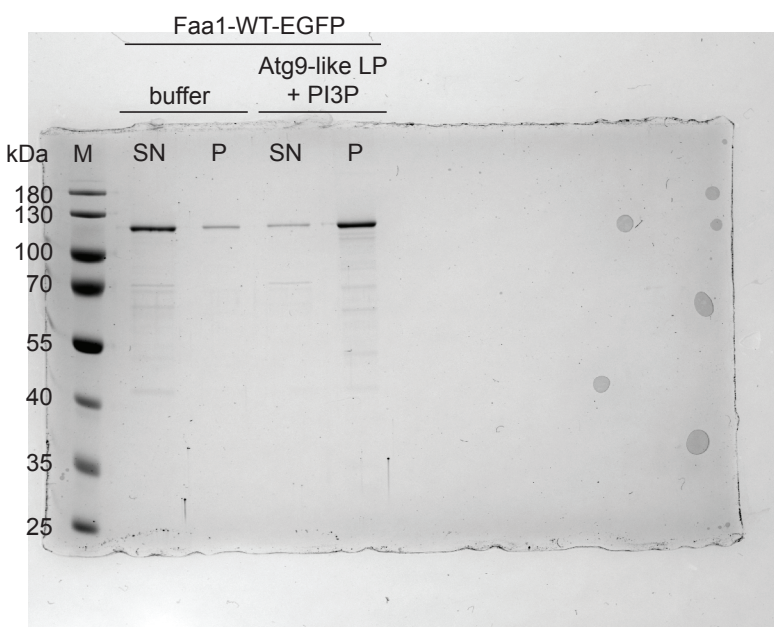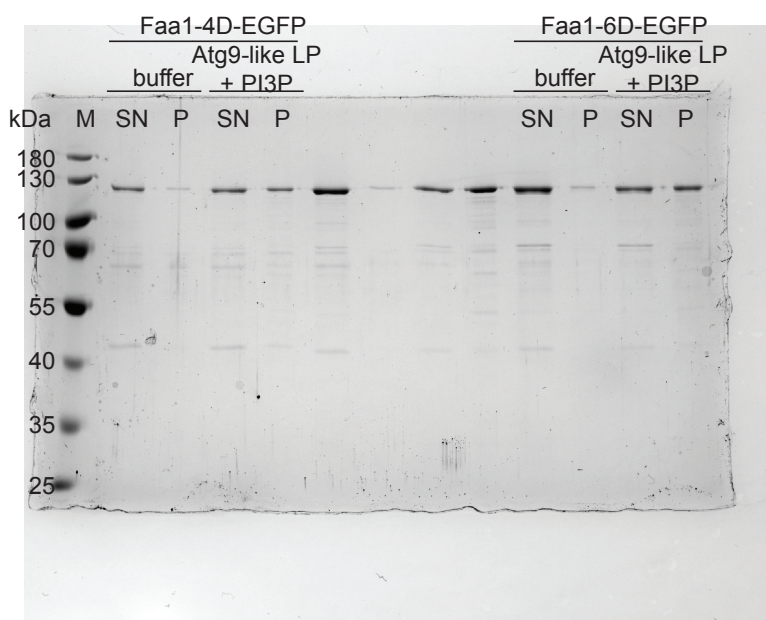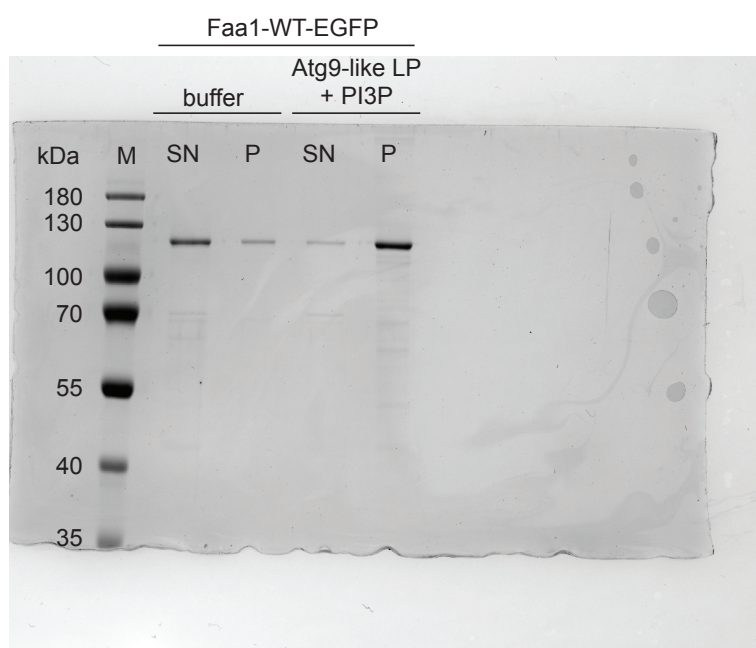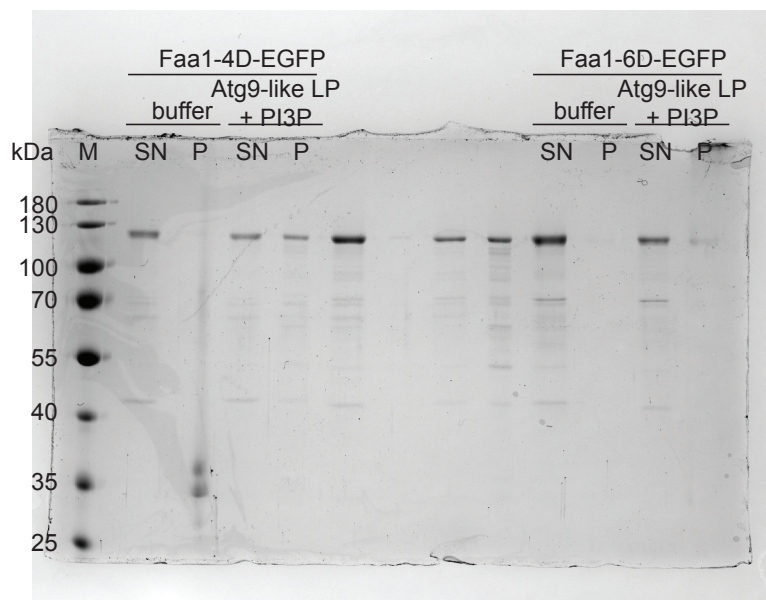

Supplement: SourceData F3 — is the source file for Fig. 3. [file JCB_202309057_SourceDataF3.pdf]

1)

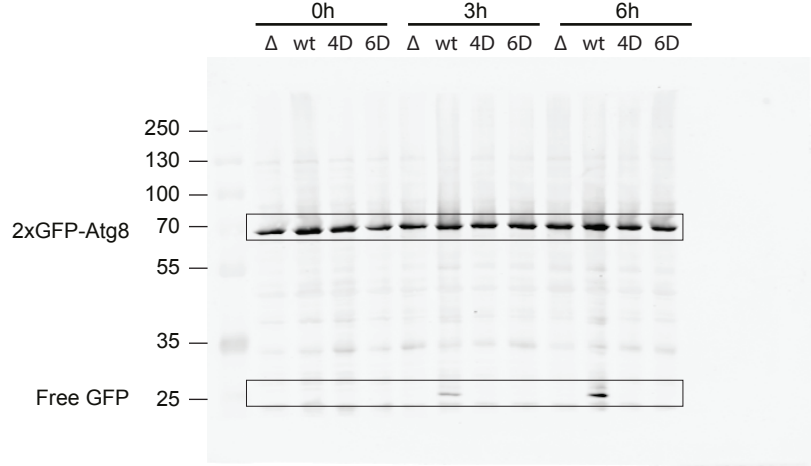

2)

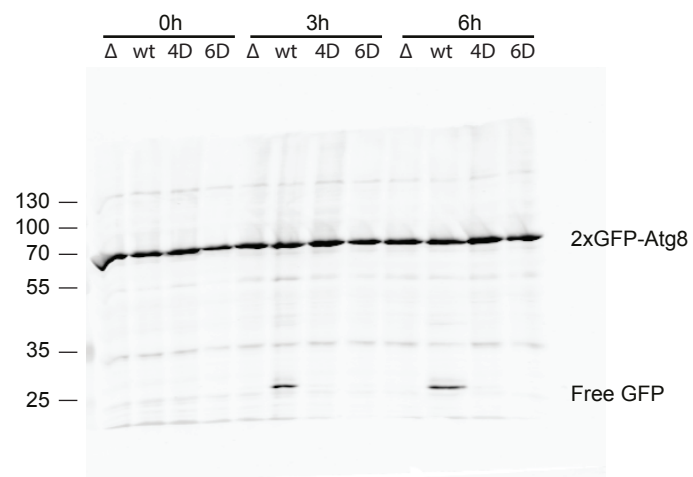

3)

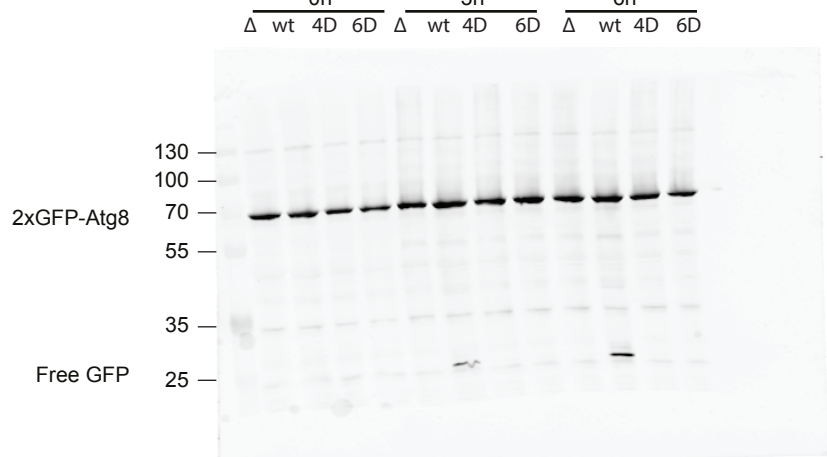

Supplement: SourceData F4 — is the source file for Fig. 4. [file JCB_202309057_SourceDataF4.pdf]

E)

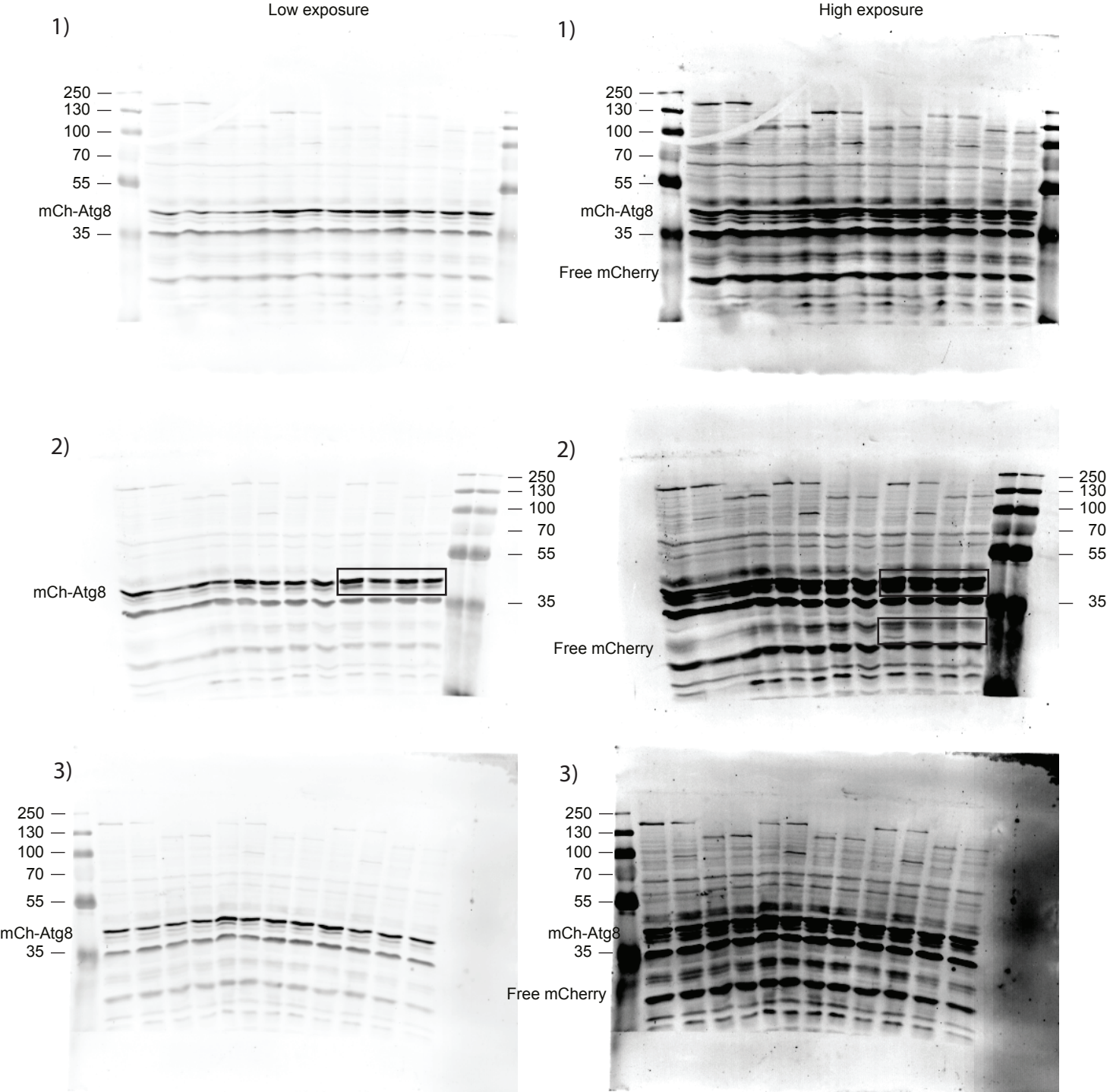

Supplement: SourceData F5 — is the source file for Fig. 5. [file JCB_202309057_SourceDataF5.pdf]

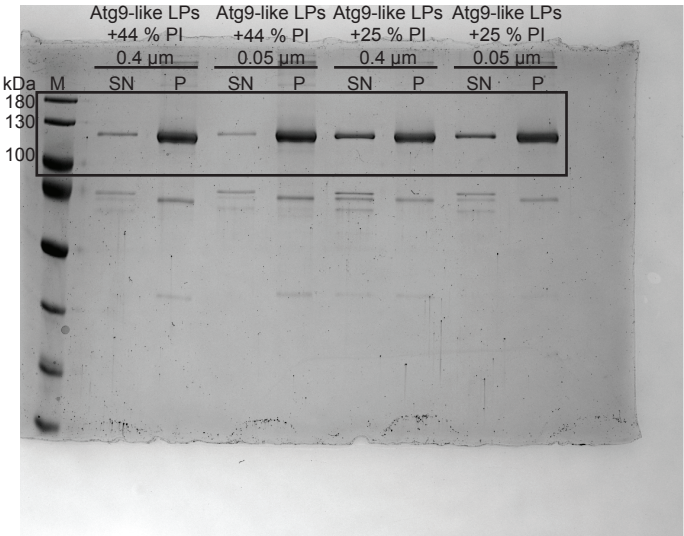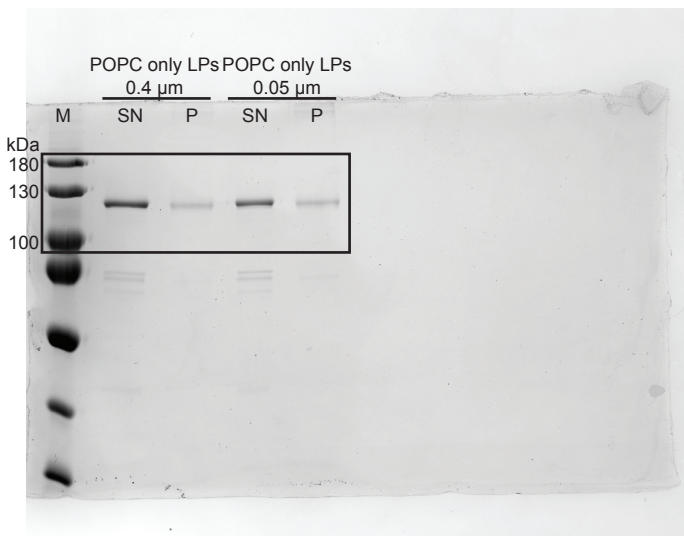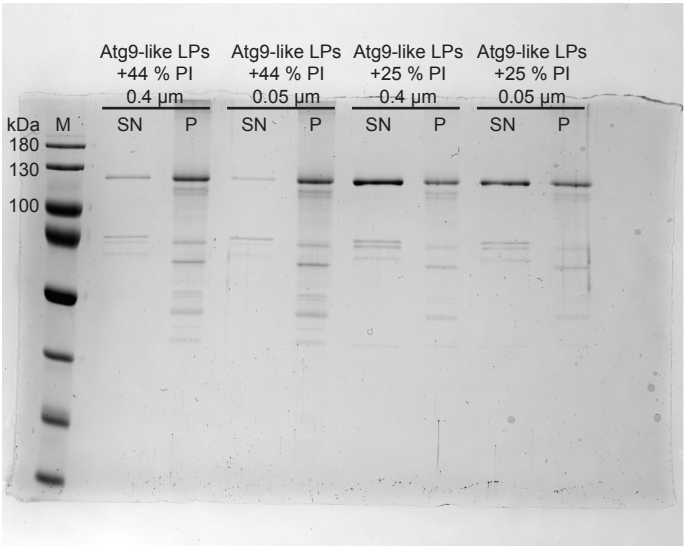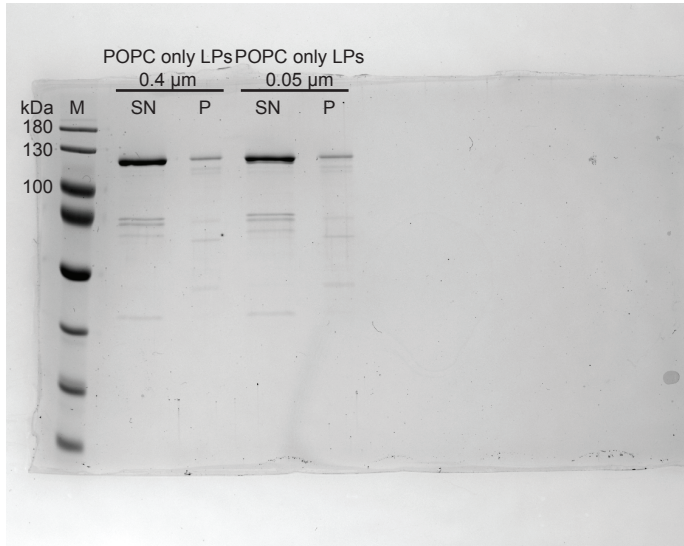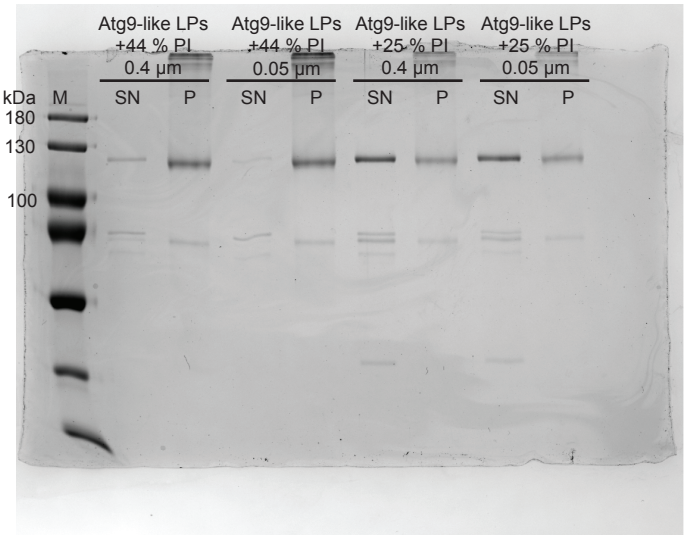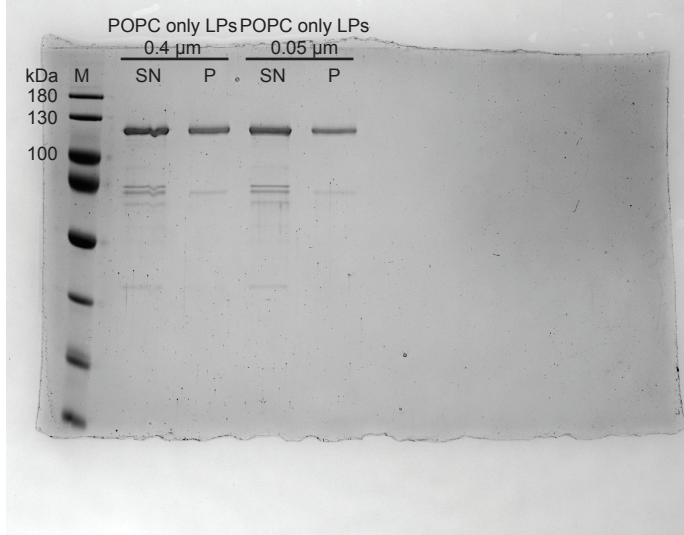

Supplement: SourceData FS1 — is the source file for Fig. S1. [file JCB_202309057_SourceDataFS1.pdf]

B)

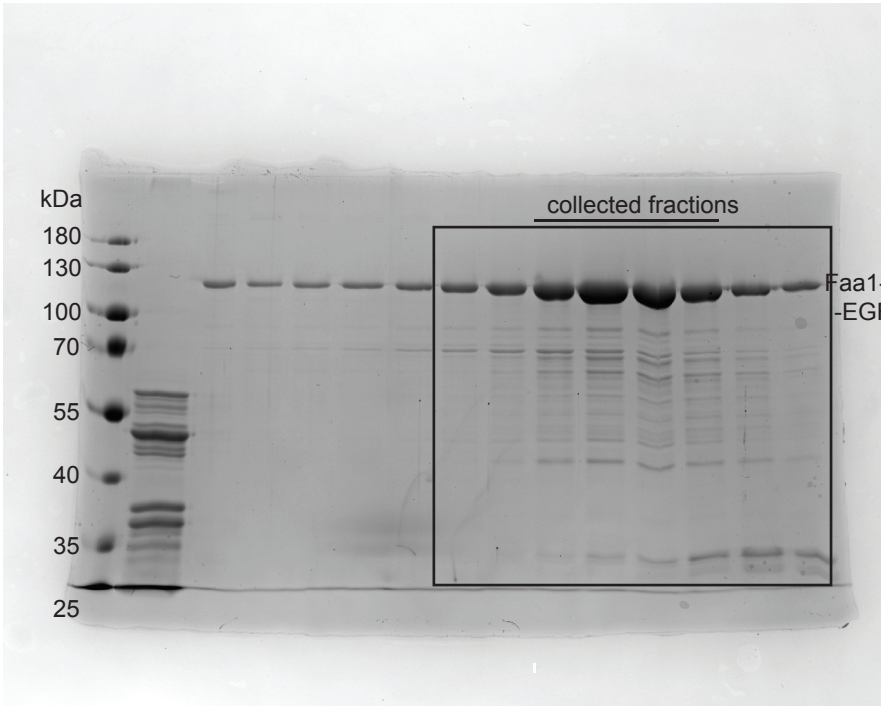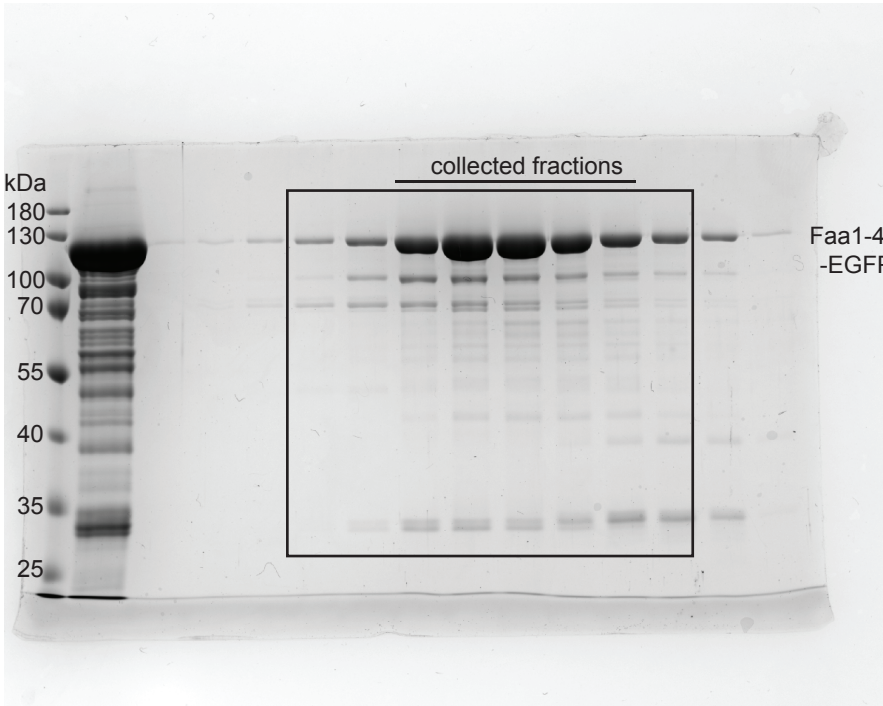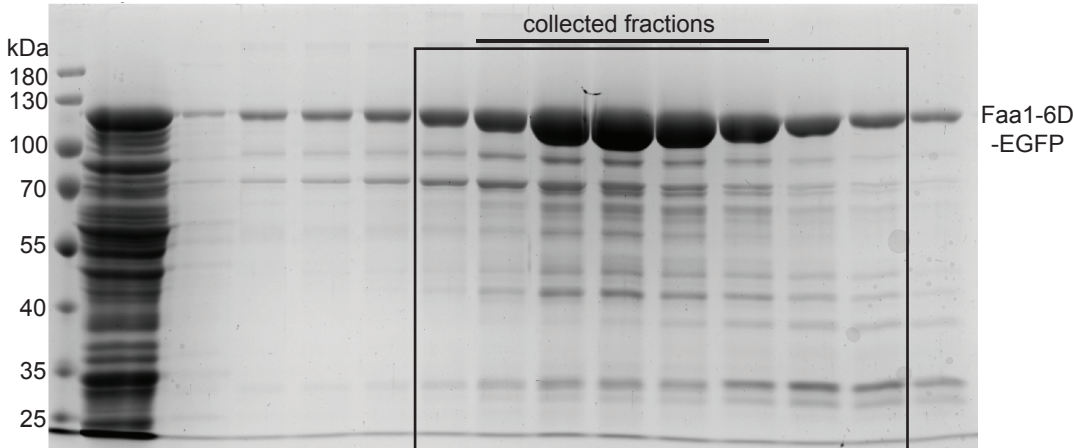

D)

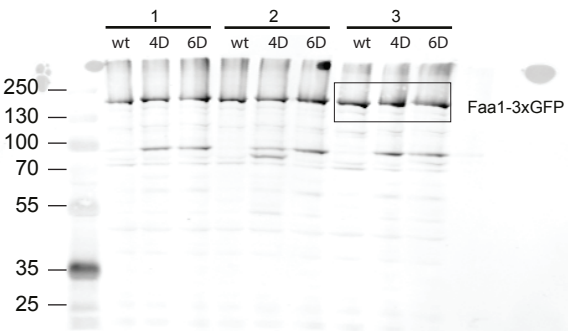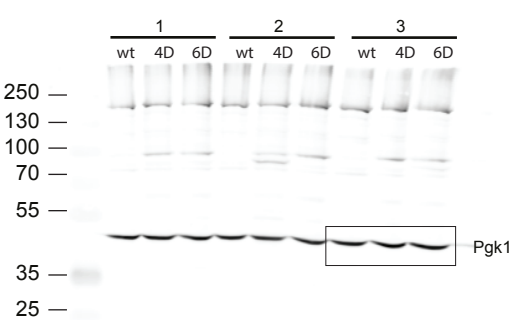

Supplement: SourceData FS2 — is the source file for Fig. S2. [file JCB_202309057_SourceDataFS2.pdf]

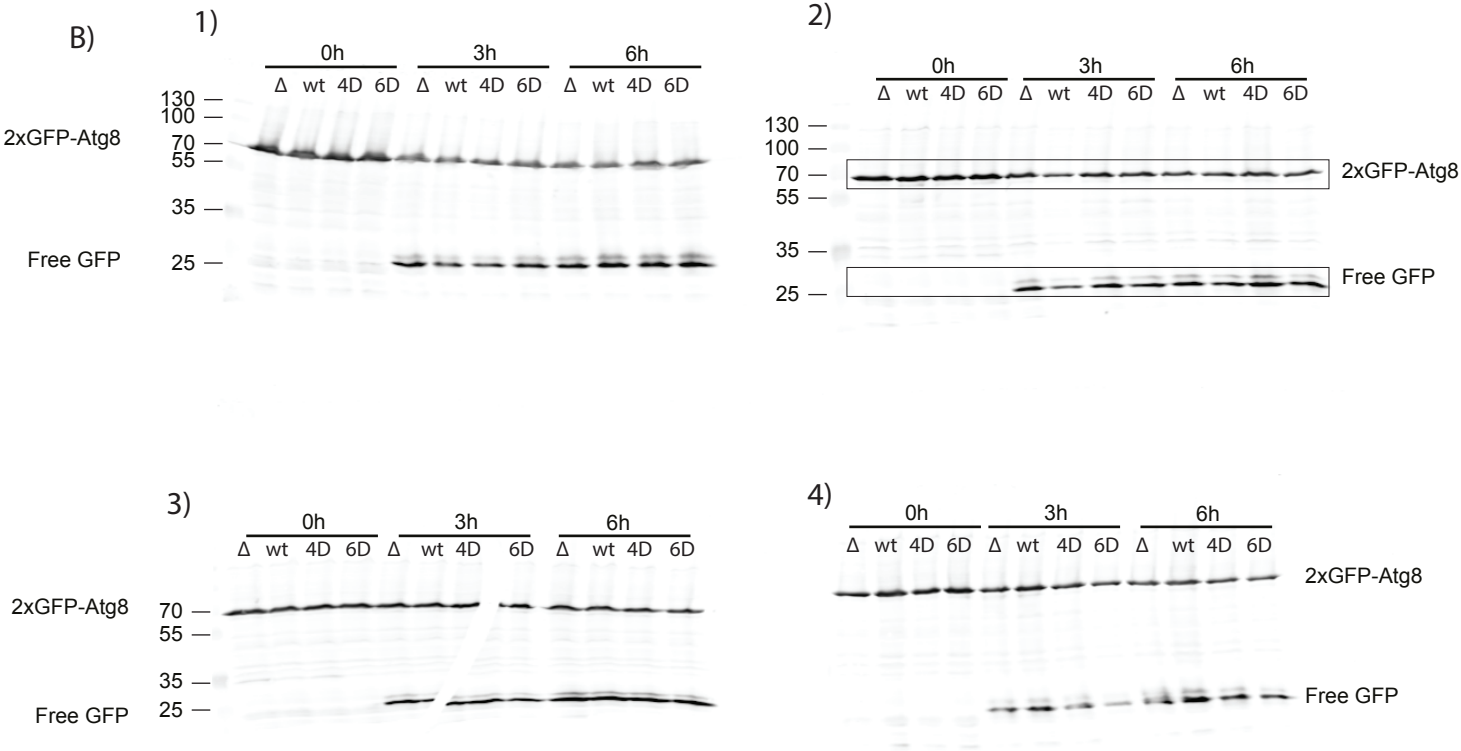

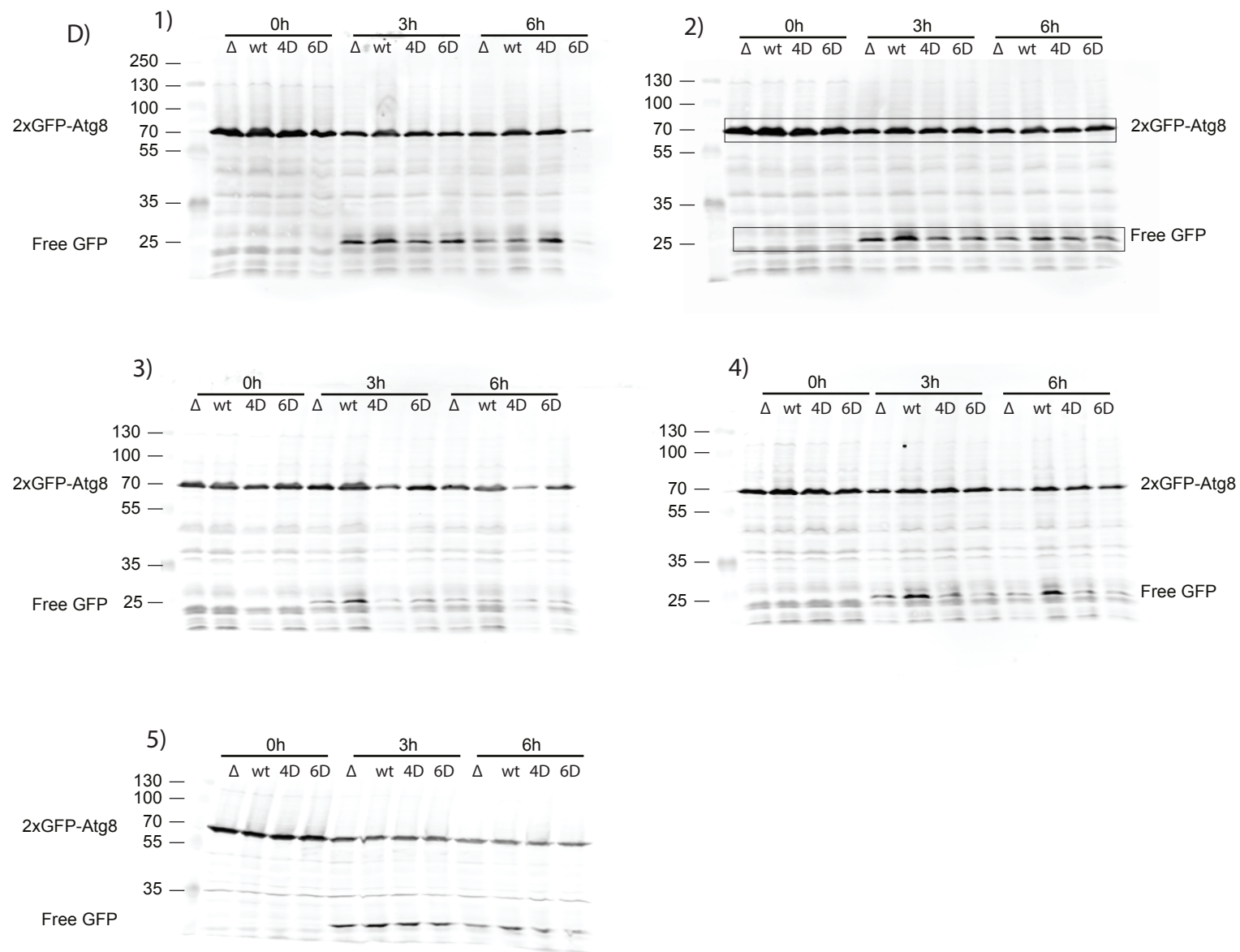



F)

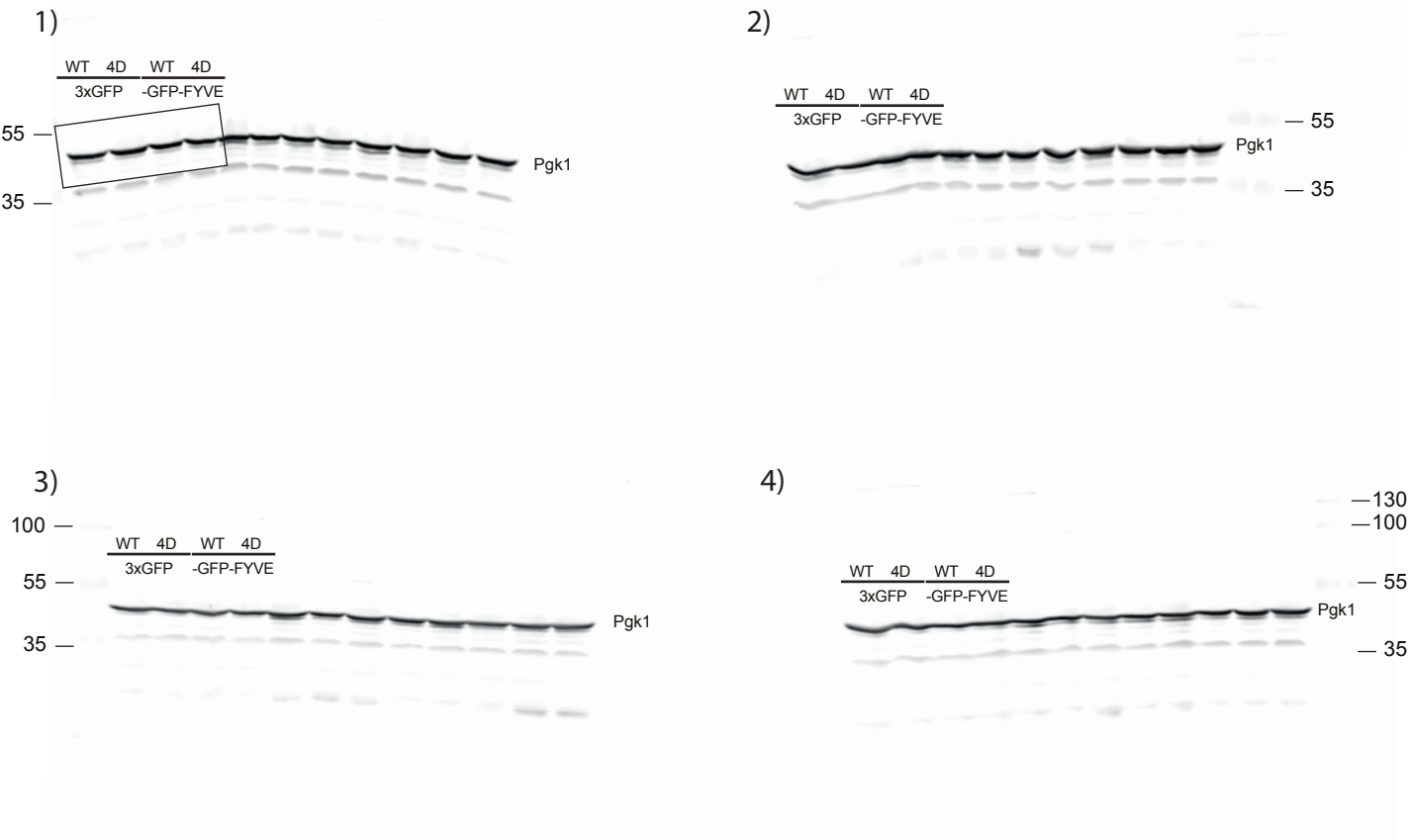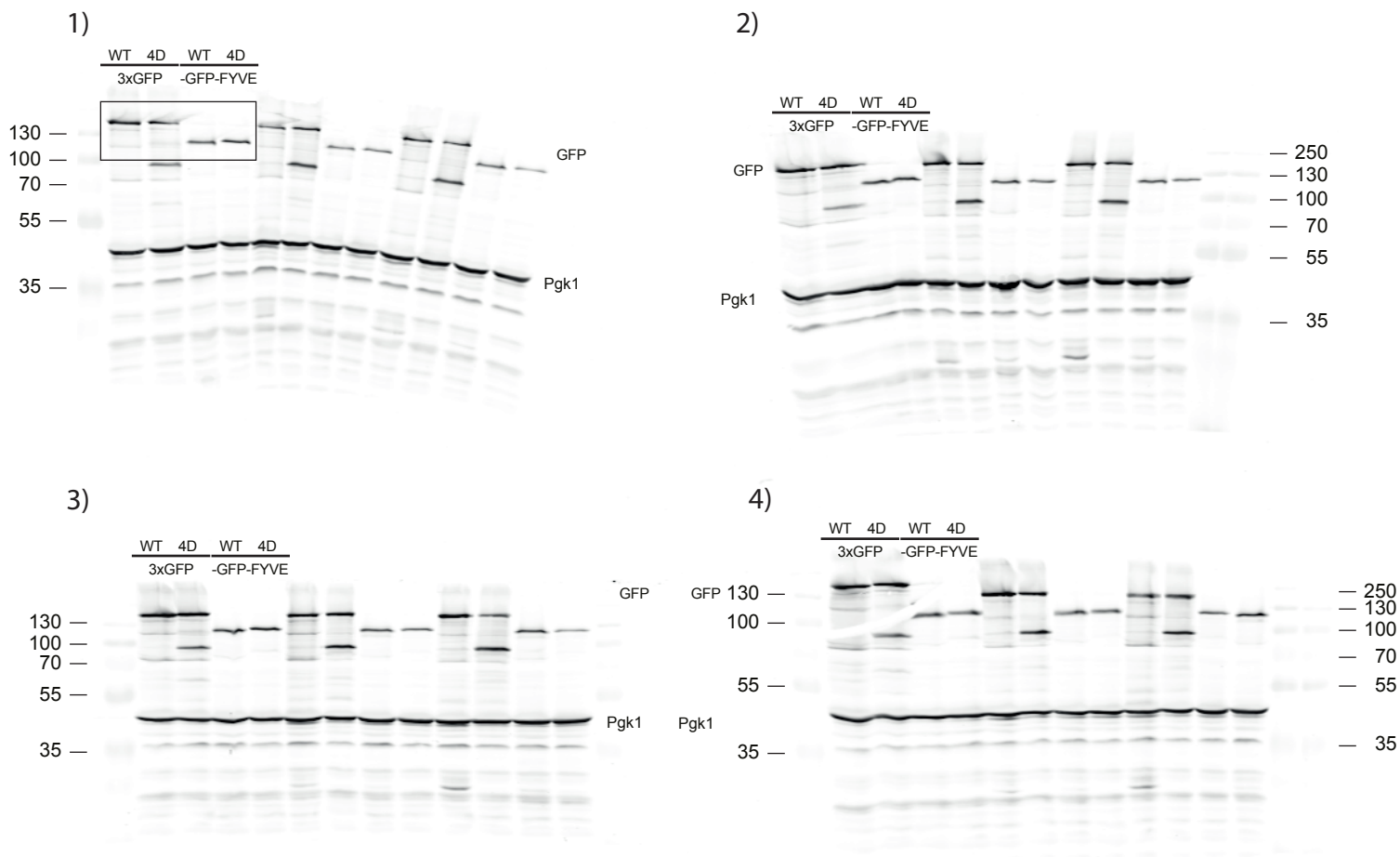

Supplement: SourceData FS3 — is the source file for Fig. S3. [file JCB_202309057_SourceDataFS3.pdf]
